# Dental pulp cell-derived powerful inducer of TNF-α comprises PKR containing stress granule rich microvesicles

**DOI:** 10.1038/s41598-019-40046-2

**Published:** 2019-03-07

**Authors:** Shigeki Suzuki, Takao Fukuda, Shintaro Nagayasu, Jun Nakanishi, Kazuma Yoshida, Shizu Hirata-Tsuchiya, Yuki Nakao, Tomomi Sano, Akiko Yamashita, Satoru Yamada, Kouji Ohta, Hideki Shiba, Fusanori Nishimura

**Affiliations:** 1 Department of Periodontology and Endodontology, Tohoku University Graduate School of Dentistry, 4-1 Seiryo-machi, Aoba-ku, Sendai 980-8575 Japan; 2 Section of Periodontology, Department of Oral Rehabilitation, Kyushu University Faculty of Dental Science, 3-1-1 Maidashi, Higashi-ku, 812-8592 Japan; 3 Department of Biological Endodontics, Graduate School of Biomedical and Health Sciences, Hiroshima University, 1-2-3 Kasumi, Hiroshima, 734-8553 Japan; 4 Department of Oral & Maxillofacial Surgery, Graduate School of Biomedical and Health Sciences, Hiroshima University, 1-2-3 Kasumi, Hiroshima, 734-8553 Japan

## Abstract

It is well known that dental pulp tissue can evoke some of the most severe acute inflammation observed in the human body. We found that dental pulp cells secrete a factor that induces tumor necrosis factor-α production from macrophages, and designated this factor, dental pulp cell-derived powerful inducer of TNF-α (DPIT). DPIT was induced in dental pulp cells and transported to recipient cells via microvesicles. Treatment of dental pulp cells with a PKR inhibitor markedly suppressed DPIT activity, and weak interferon signals were constitutively activated inside the cells. In recipient macrophages, stimulation with DPIT-containing supernatants from pulp cells resulted in activation of both nuclear factor-κB and MAP kinases like JNK and p38. Proteomics analyses revealed that many stress granule-related proteins were present in supernatants from dental pulp cells as well as microvesicle marker proteins like GAPDH, β-actin, HSPA8, HSPB1, HSPE1, and HSPD1. Furthermore, giant molecule AHNAK and PKR were detected in microvesicles derived from dental pulp cells, and gene silencing of AHNAK in dental pulp cells led to reduced DPIT activity. Thus, it appeared that the core protein of DPIT was PKR, and that PKR was maintained in an active state in stress granule aggregates with AHNAK and transported via microvesicles. The activity of DPIT for TNF-α induction was far superior to that of gram-negative bacterial endotoxin. Therefore, we, report for the first time, that active PKR is transported via microvesicles as stress granule aggregates and induces powerful inflammatory signals in macrophages.

## Introduction

Dental pulp cells are continuously exposed to various environmental stresses such as hot and cold temperatures, mechanical stress, and bacterial irritation^1^. Once acute inflammation has been evoked in dental pulp tissue, the inflammation is rapidly up-regulated inside the tooth and the tissue usually undergoes complete necrosis within a few days^1^. However, the exact mechanism for the establishment of this acute, severe inflammatory reaction is not fully understood.

**Figure 1:**
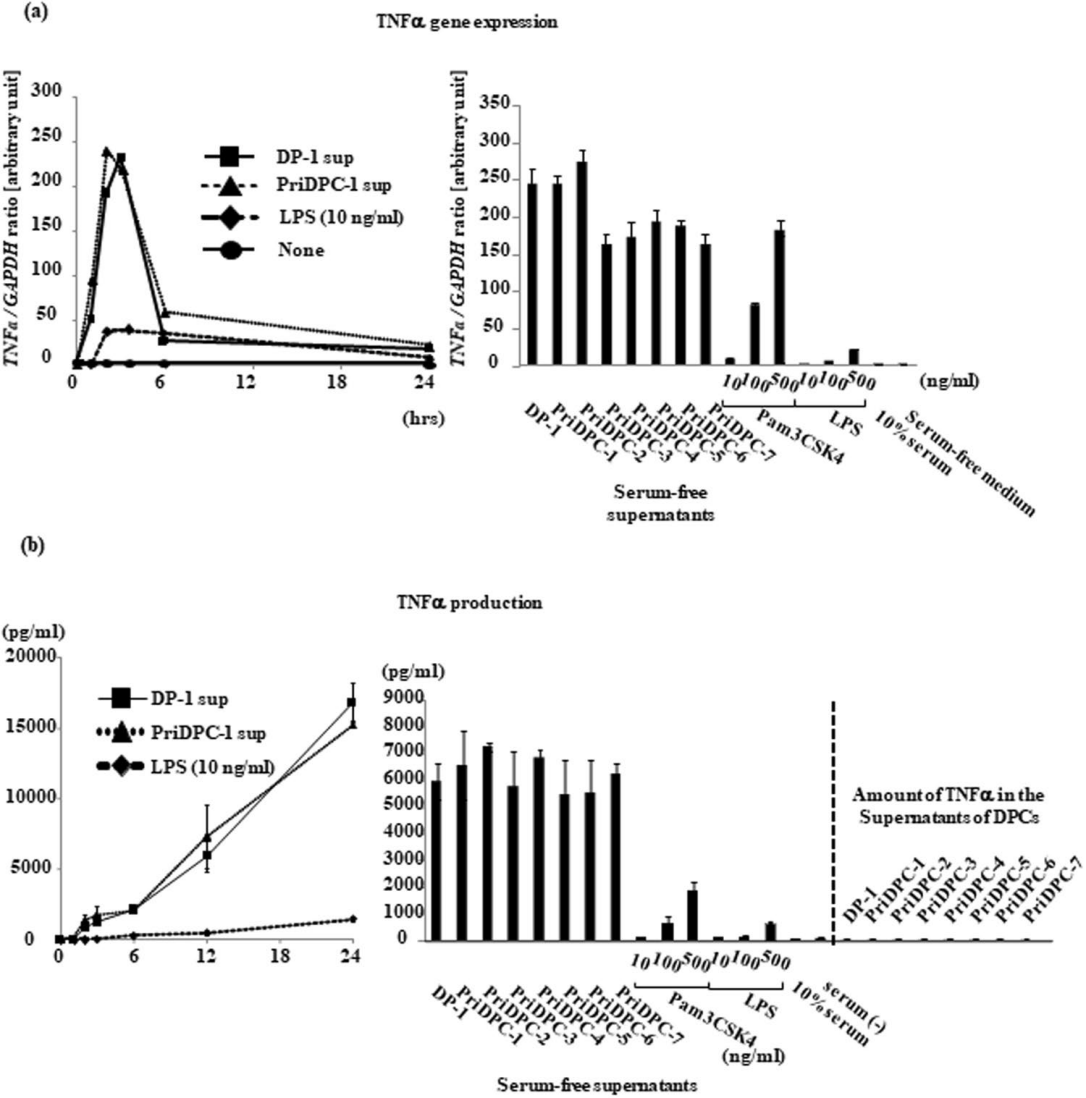
DPIT is a powerful TNF-α stimulator. (**a**) TNF-α gene expression and (**b**) TNF-α production was examined in differentiated THP-1 (dTHP-1) cells stimulated with the supernatants from DP-1 and primary dental pulp cells (priDPC)-1. dTHP-1 cells are stimulated with the supernatants from DP-1 (DP-1 sup) and priDPC (priDPC sup), LPS or Pam3CSK4 at indicated concentration for indicated time periods, and total RNAs and supernatants are collected. (**a**: right) dTHP-1 cells are stimulated for 2 hrs with various stimulants and gene expression was analyzed by qPCR. (**b**: right) Culture supernatants of dTHP-1 cells were collected 2 hrs after stimulation and TNF-α protein level was measured by ELISA. Amounts of TNF-α in culture supernatants of original dental pulp cells were also measured (**b**: right).

**Figure 2:**
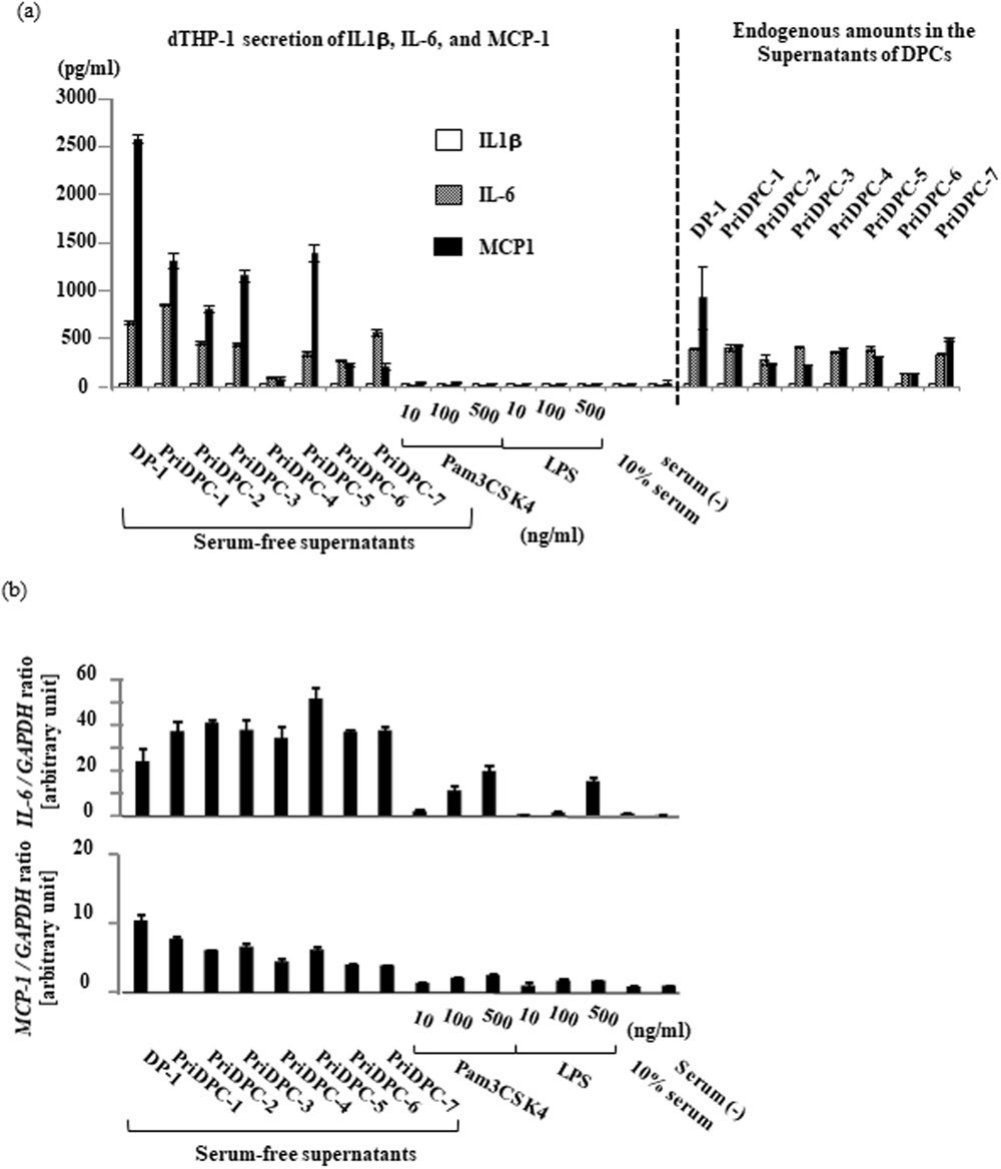
DPIT also induces IL-6 and MCP-1 expression in differentiated THP-1 cells. (**a**) The production of IL-1β, IL-6, and MCP-1, and (**b**) the gene expression of IL-6 and MCP-1 (**b**) in differentiated THP-1 (dTHP-1) cells stimulated with the supernatants from DP-1 and primary dental pulp cells (priDPC) were evaluated. dTHP-1 cells are stimulated with the supernatants from DP-1 (DP-1 sup) and priDPC (priDPC sup), LPS or Pam3CSK4 at indicated concentration, and total RNAs and supernatants are collected. Culture supernatants of dTHP-1 cells were collected 2 hrs after the stimulation and IL-1β, IL-6, and MCP-1 protein levels were measured by ELISA. For gene expression analysis, dTHP-1 cells were stimulated with various stimulants for 2 hrs. Endogenous amounts of these cytokines in culture supernatants of dental pulp cells were also measured (**a**).

**Figure 3:**
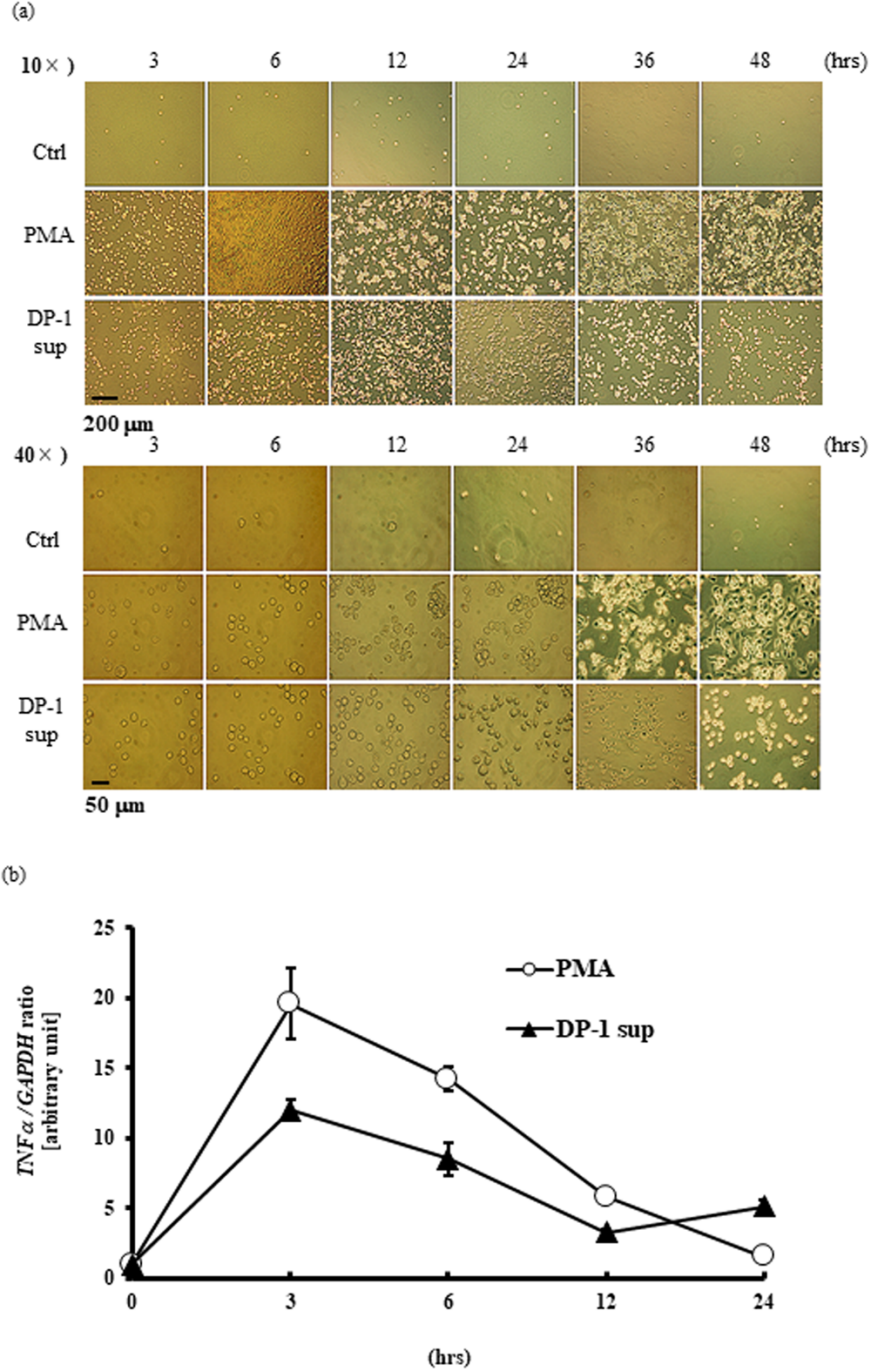
DPIT also stimulates undifferentiated THP-1 cells. Floating undifferentiated THP-1 (unTHP-1) cells were stimulated with DP-1 sup or PMA for indicated time periods and cell attachment was visualized with phase-contrast microscopy after non-attached cells are washed out (**a**). DP-1 sup promoted cell attachment to the culture plate as PMA. Simultaneously, total RNAs are also collected to see the TNF-α gene expression in unTHP-1 cells stimulated with DP-1 sup or PMA (**b**). Both DP-1 sup and PMA promoted TNF-α gene expression.

**Figure 4:**
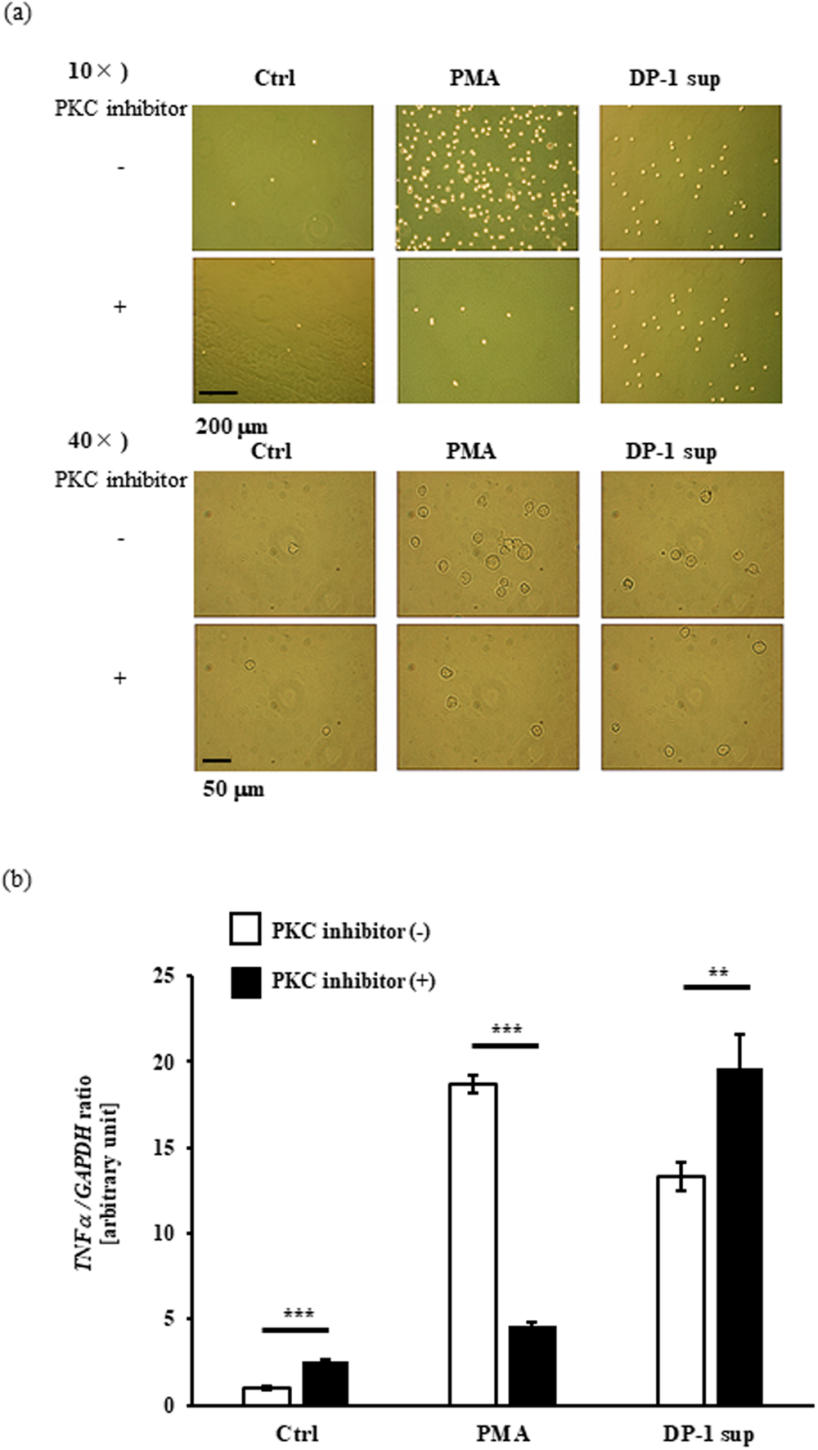
THP-1 cell activation by DPIT is independent of PKC activation. (**a**) Floating unTHP-1 cells were stimulated for 3 hrs with DP-1 sup or PMA with a PKC inhibitor (GF109203X: 10 μM) and cell attachment was visualized with phase-contrast microscopy. (**b**) Simultaneously, total RNAs are collected to see TNF-α gene expression (**b**). Although PKC inhibitor markedly suppressed unTNP-1 cell attachment to the culture plate and TNF-α gene expression in unTHP-1 cells in the presence of PMA, no inhibitory effect was observed when the cells were stimulated DP-1 sup. Data represents means ± SD of at least two independent experiments with identical results. Statistical analyses were performed by Student’s *t*-test. **p < 0.01, ***p < 0.001, significantly difference versus cells untreated with PKC inhibitor.

We found that dental pulp cells, both immortalized cells^2^ and primary cells, secrete a factor that strongly stimulates differentiated THP-1 (dTHP-1) cells to induce tumor necrosis factor (TNF)-α at both the gene and protein levels (Fig. 1a: left, 1b: left), and designated this factor dental pulp cell-derived powerful inducer of TNF-α (DPIT). DPIT activity was seen in both immortalized dental pulp cells (DP-1) and primary dental pulp cells (PriDPC) and the activity was superior to that of a gram-negative bacterial endotoxin, lipopolysaccharide (LPS) when dTHP-1 cells were incubated for 2 hrs (Fig. 1a: right, 1b: right). Culture supernatants from dental pulp cells also stimulated dTHP-1 cells to express genes and secrete interleukin (IL)-6 and monocyte chemoattractant protein (MCP)-1 proteins (Fig. 2a,b). Because small amounts of IL-6 and MCP-1 were detected in culture supernatants from DP-1 and PriDPCs, we examined the possibility that cytokine-stimulated dTHP-1 cells may be induced toward TNF-α expression. However, these cytokines did not induce TNF-α expression in dTHP-1 cells even after the 24-hr incubation (Supplementary Fig. 1). Furthermore, IL-1β and IL-32, a potent TNF-α inducer from macrophages^3,4^, was not detected in culture supernatants of DP-1 and PriDPCs (data not shown). We therefore considered that DPIT could be a novel pro-inflammatory factor. Interestingly, culture supernatants from DP-1 and PriDPCs appeared to accelerate cell attachment to culture dishes in undifferentiated floating THP-1 cells (Fig. 3a), similar to phorbol 12-myristate 13-acetate (PMA) stimulation, and also induced TNF-α gene expression in undifferentiated THP-1 cells (Fig. 3b). Moreover, DP-1 supernatant, but not PMA, induced proliferative property for adhered THP-1 cells (Supplementary Fig. 2). Therefore, it can be concluded that this pro-inflammatory factor from dental pulp cells, DPIT, is a universal activator of monocytic cells. In general, phorbol esters like PMA, which is frequently used for differentiation of THP-1 monocytic cells into macrophages^5^, mimic the effect of diacylglycerol, and lead to activation of protein kinase C (PKC) in target cells^6^. However, although a PKC inhibitor suppressed PMA-induced THP-1 cell adhesion and subsequent TNF-α gene expression, no inhibitory effect of the PKC inhibitor on DP-1 supernatant was observed with respect to both cell attachment and TNF-α gene expression (Fig. 4a,b), indicating that DPIT activity is exerted by a mechanism independent of PKC activation.

**Figure 5:**
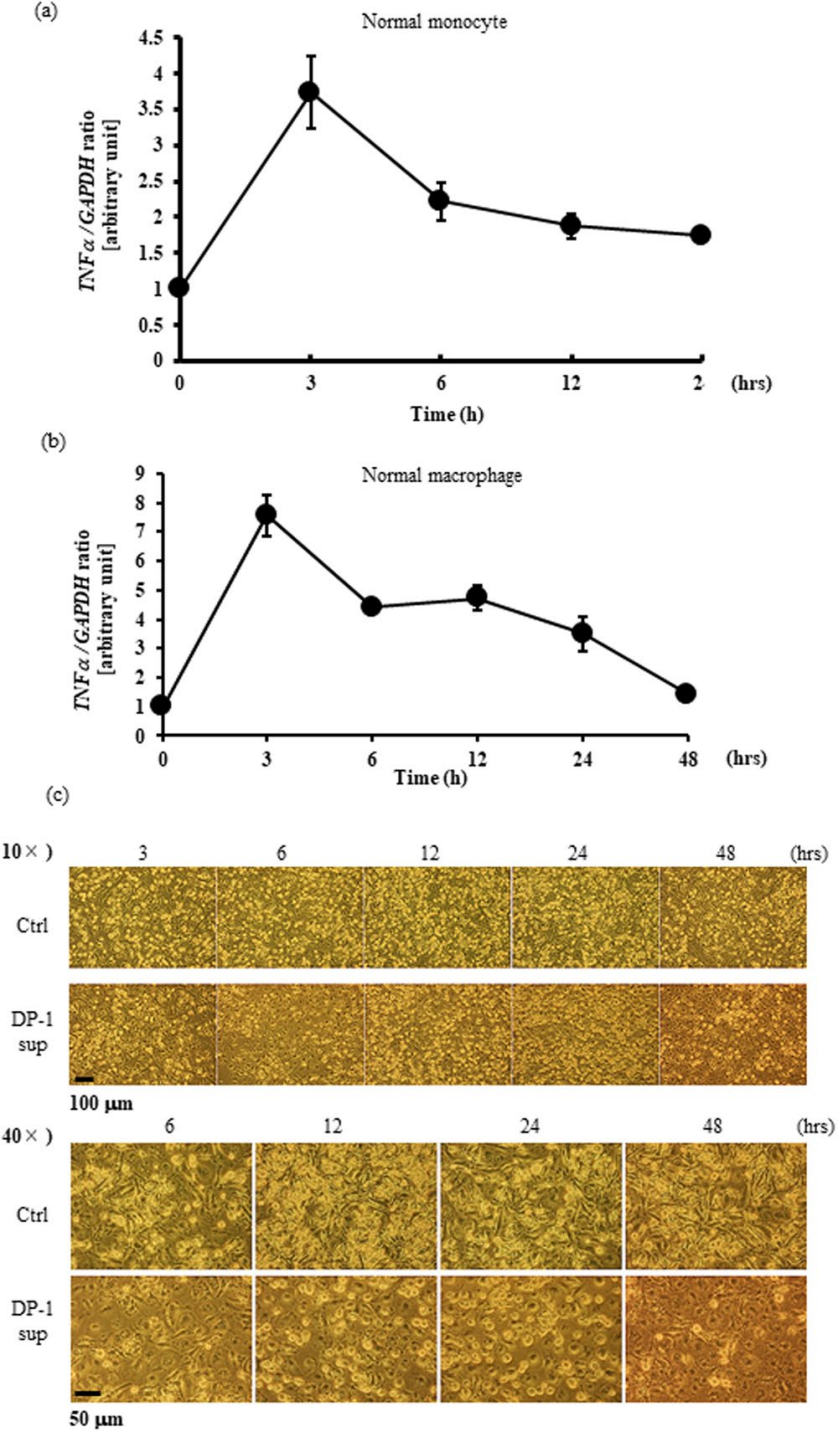
DPIT also activates normal human monocytes and macrophages. (**a**,**b**) Induction of TNF-α gene expression in human normal CD14-positive monocytes (**a**) and differentiated macrophages induced by M-CSF from PBMC (**b**). These cells were stimulated with DP-1 sup for indicated time periods and total RNAs were collected. (**c**) Induction of morphological changes of normal human macrophages to a “fried egg-like” appearance. Differentiated normal human macrophages were stimulated with DP-1 sup for indicated time periods and adhered cells were visualized with phase-contrast microscopy.

Next, we examined the effects of DP-1 supernatants on normal human monocytes and macrophages. The DP-1 supernatant was found to induce TNF-α gene expression in normal human monocytes like THP-1 cells (Fig. 5a). Furthermore, the DP-1 supernatant was able to not only stimulate differentiated macrophages from CD14^+^ monocytes by M-CSF to induce TNF-α gene expression (Fig. 5b), but also promote morphological changes from s spindle shape to a fried-egg shape (Fig. 5c)^7^. Taken together, we confirmed that culture supernatants from dental pulp cells contained a factor that could strongly induce pro-inflammatory reactions.

Thus, we, aimed to clarify the nature of this pro-inflammatory factor, designated DPIT. To determine the differences in gene expression between cells with and without DPIT activity, we compared gene expression profiles between DP-1 with DPIT activity and gingival tissue-derived fibroblasts without DPIT activity, and between PriDPC-1 with DPIT activity and gingival tissue-derived fibroblasts without DPIT activity by DNA microarray analyses (the entire DNA microarray data base is deposited at www.ncbi.nlm.nih.gov/geo, GSE124492). We selected genes that were commonly up-regulated in both DP-1 and PriDPC-1 associated with high DPIT activity (Table 1). Interestingly, many of the commonly up-regulated genes were family members of interferon-inducible genes, suggesting that dental pulp cells were constantly exposed to weak stress stimuli. Interferon signals are known to activate interferon-induced double-stranded RNA-activated protein kinase (EIF2AK2), also known as protein kinase R (PKR)^8^. In fact, we found in our microarray analyses that PKR expression was elevated by in DP-1 and PriDPC-1, respectively, compared with control gingival fibroblasts and the significant up-regulation of PKR expression in both DP-1 and PriDPC-1 was further confirmed by qPCR analysis utilizing the primer pairs targeting for PKR (Supplementary Fig. 3).

**Table 1:** Gene list with higher expression in DPTIF-positive cells.

priDPC>GF	common	DP-1>GF
MX1	MX1	CXCL1
TFPI2		CXCL1
CXCL1	CXCL1	RPL28
LOC645638		CSF2
LOC645638		TMEM203
IFIT2	IFIT2	OASL
IFIT2	IFIT2	IFI27
SRGN		PITX1
MX2	MX2	CXCL2
SMCR7L	SMCR7L	IL8
KRTAP1-5		CD70
LZTS1		LOC285628
IFIT3	IFIT3	CXCL3
NPM2		
BAMBI		OAS1
OAS2	OAS2	CXCL6
OAS3	OAS3	C3
CTGF		APOBEC3B
LOC400550		PRUNE2
NPR3		CSF3
CCL2		MX1
CCL2		SAA2
CCL2		CXCL2
CCL2		HOXB6
CCL2		PDLIM3
IFIT1	IFIT1	C4orf7
CCL2		RSAD2
CCL2		PNMA2
ERAP2	ERAP2	C3
CCL2		INPP5D
CNN1		NFIB
BATF2	BATF2	TNNT1
CCL2		CLMN
CCL2		HLA-DOA
RAB11FIP1		APOC1
IL6	IL6	GPAT2
SLC2A1		IL1B
IFI6	IFI6	NGEF
EPSTI1	EPSTI1	C1QL4
KRTAP1-5		EYA4
EIF2AK2 (PKR)	EIF2AK2 (PKR)	EIF2AK2 (PKR)

**Figure 6:**
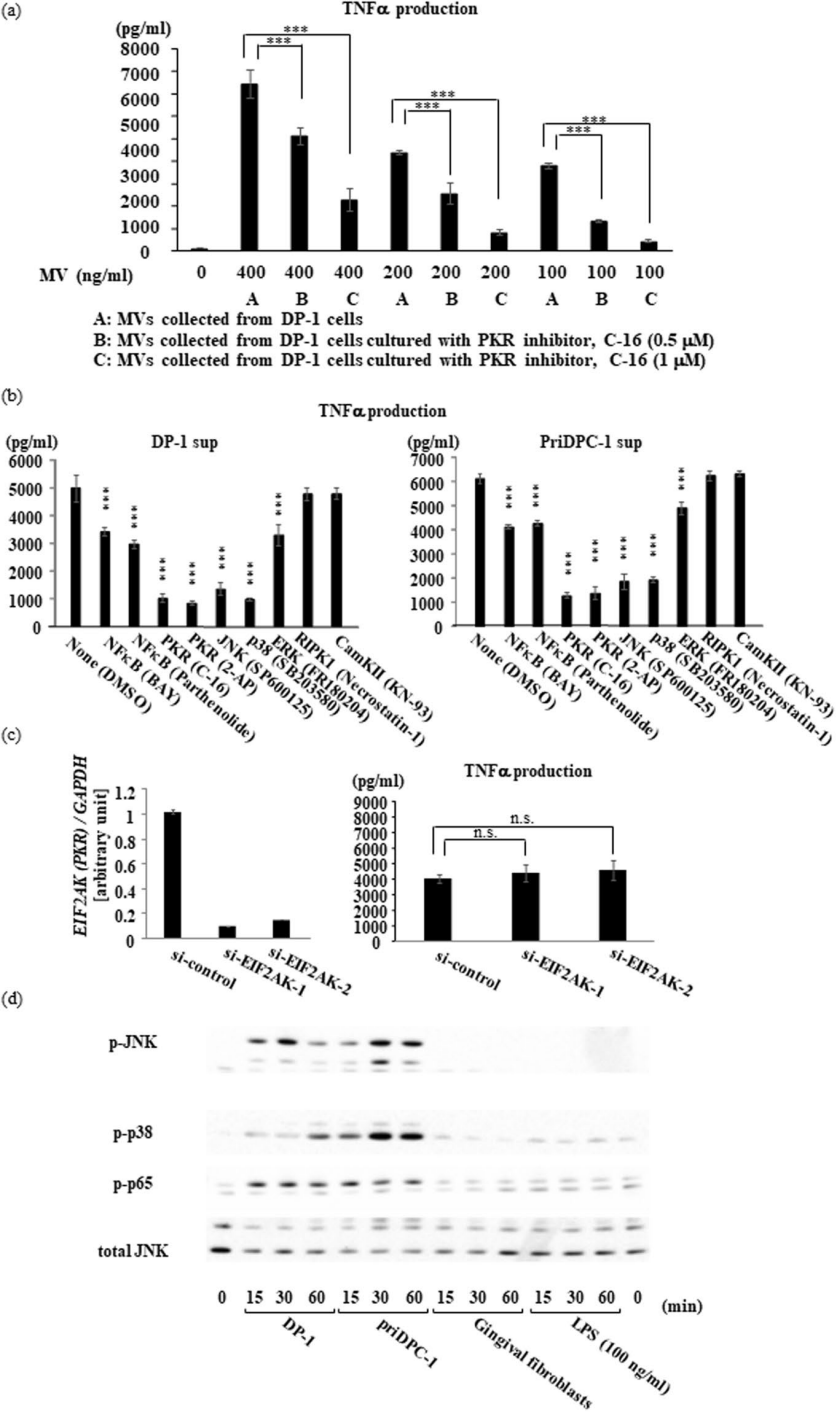
PKR is the primary molecule exhibiting DPIT activity. (**a**) Microvesicles (MVs) purified from DP-1 sup possessed the ability to induce TNF-α secretion in dTHP-1 cells, and treatment of DP-1 cells with PKR inhibitor (C-16) reduced the TNF-α-inducible ability of these MVs. DP-1 cells were treated with C-16 (0, 0.5, or 1 μM) for 24 hrs and then MVs were purified from the supernatants. Then, dTHP-1 cells were stimulated with the various concentrations of these purified MVs for 2 hrs and the amounts of secreted TNF-α were quantified. (**b**) Pre-treatment of dTHP-1 cells with a PKR inhibitor (C-16: 1 μM, 2-AP: 20 mM) also suppressed TNF-α production after stimulation with DP-1 sup and PriDPC-1 sup. Note that pre-treatment with inhibitors of NFκB (BAY 11-70: 10 μM; Parthenolide: 1 μM), JNK (SP600125: 10 μM), p-38 SB203580: 10 μM), and ERK (FR180204: 10 μM) also suppressed TNF-α production, while pre-treatment with inhibitors of RIPK1 (necrtostatin-1: 10 μM) and CamKII (KN-93: 10 μM) did not alter TNF-α production. (**c**) In contrast, treatment of dTHP-1 cells with siRNAs to successfully down-regulate endogenous PKR (left) did not influence TNF-α production after stimulation with supernatants from DP-1 (right). (**d**) Culture supernatants from dental pulp cells (DP-1, priDPC-1), but not those from gingival fibroblasts, markedly activated JNK, p38, and NF-κB in dTHP-1 cells. The ability of DP-1 sup and PriDPC-1 sup in cell activation is extremely powerful as compared with that of LPS (right). n.s.: not significant. Data represent means ± SD of at least two independent experiments with identical results. Statistical analyses were performed by one-way ANOVA, followed by Tukey’s test (**a**,**c**) or Dunnett’s test (**b**). ***p < 0.001, significant differences versus dTHP-1 cells treated with equivalent amounts of MVs from non-treated dDP-1 cells (**a**) or dTHP-1 cells treated with DMSO (b).

It is very unlikely that PKR alone is transported extracellularly from dental pulp cells to recipient macrophages. We, therefore, hypothesized that PKR may be transported from dental pulp cells to macrophages via extracellular vesicles. To test this possibility, we collected extracellular vesicles from DP-1 treated with a PKR inhibitor, and examined their DPIT activity. We found that DPIT activity was inhibited after treatment of DP-1 with the PKR inhibitor in a dose-dependent manner (Fig. 6a). Pre-incubation of dTHP-1 cells with PKR inhibitors also suppressed TNF-α production in dTHP-1 cells following dental pulp cell-derived supernatant stimulation (Fig. 6b). Interestingly, however, down-regulation of endogenous PKR in dTHP-1 cells by small interfering RNA (siRNA) transfection did not decrease TNF-α production after stimulation with DP-1 supernatant (Fig. 6c), suggesting that TNF-α expression was not a result of endogenous PKR activation in recipient macrophages. Culture supernatants from dental pulp cells, both DP-1 and PriDPC-1, markedly induced phosphorylation of p38 MAP kinase (p38), c-Jun N-terminal kinase (JNK), and nuclear factor (NF)-κB in dTHP-1 cells (Fig. 6d). LPS-dose-dependent phosphorylation of these kinases has been confirmed to verify the validity and quality of THP-1 cells used in this study (Supplementary Fig. 4). Because PKR was reported to activate p38, JNK and NF-κB^8–10^, we hypothesized that DPIT activity was mediated by PKR derived from dental pulp cells, and that the possible transport machinery was extracellular particles.

**Figure 7:**
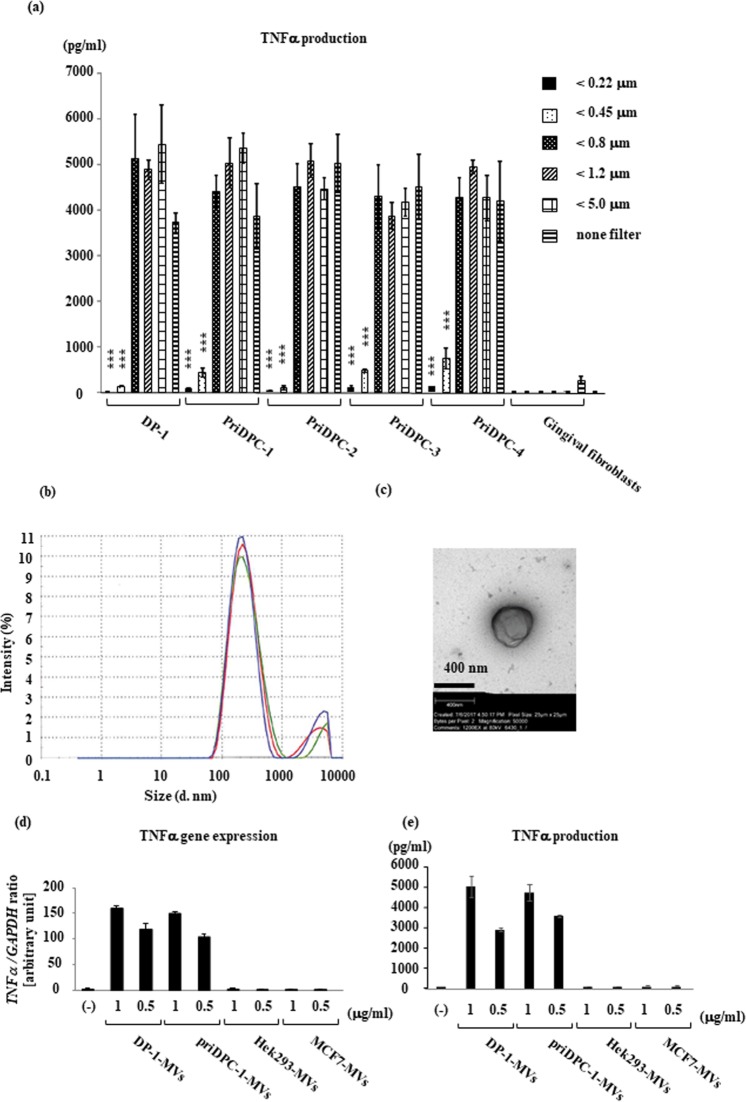
PKR is transported from dental pulp cells via microvesicles. (**a**) Size exclusion of MVs from the supernatants of dental pulp cells with a pore size below 220 nm completely abolished DPIT activity. (**b**) The most abundant vesicles are 230–250 nm in size by Dynamic light scattering measurements. (**c**) Representative MVs photographed by TEM analysis are shown. (**d**,**e**) DP-1-MVs and PriDPC-1-MVs up-regulated TNF-α gene expression (**d**) and protein production (**e**). Data represent means ± SD of at least two independent experiments with identical results. Statistical analyses were performed by one-way ANOVA, followed by Tukey’s test. ***p < 0.001. significant difference versus dTHP-1 cells stimulated with non-filtered supernatants.

**Figure 8:**
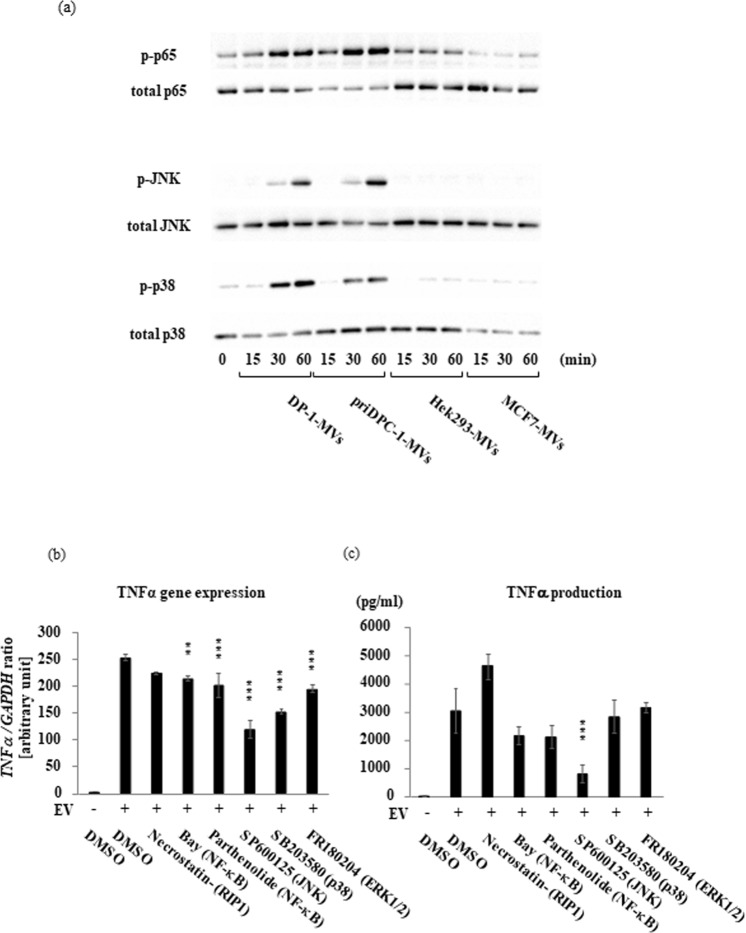
Dental pulp cell-derive MVs activates NF-*κ*B and MAP kinase (MAPK) in dTHP-1 cells to produce TNF-α. (**a**) DP-1-MVs, priDPC-1-MVs, but not Hek293-MVs and MCF7-MVs activated JNK and p38 MAPK in dTHP-1 cells. (**b**) NF-κB and MAPK, especially JNK, are required for DP-1-MVs-induced TNF-α gene expression (left) and protein production (right) in dTHP-1 cells, while necrostatin treatment to suppress RIP-1 had no effect. Data represent means ± SD of at least two independent experiments with identical results. Statistical analyses were performed by one-way ANOVA, followed by Tukey’s test. **p < 0.01, ***p < 0.001. significant difference versus dTHP-1 cells treated with DMSO before stimulation with MVs (**b**,**c**).

**Figure 9:**
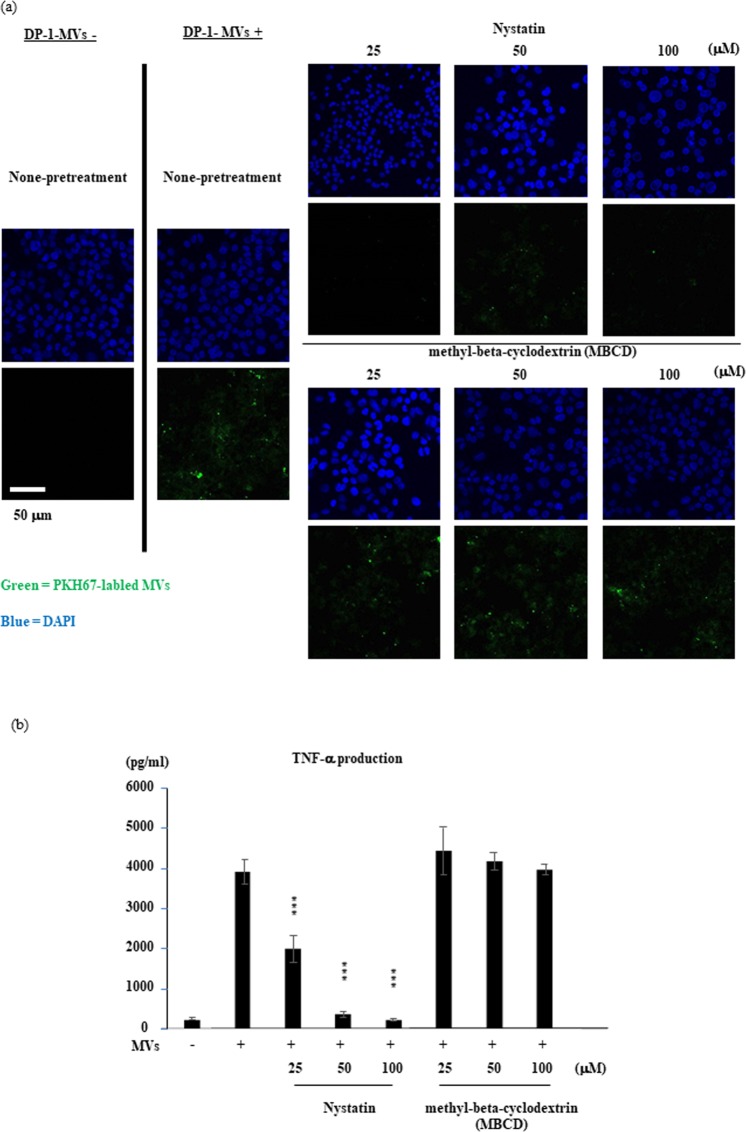
Uptake of dental pulp cell-derived MVs by recipient dTHP-1 cells. (**a**,**b**) DP-1-MVs uptake triggers TNF-α production in dTHP-1 cells. PKH67-labeled DP-1-MVs are incorporated by dTHP-1 cells (**a**), while cellular uptake (**a**) and TNF-α production (**b**) are suppressed by pre-treatment of dTHP-1 cells with nystatin, but not MBCD (**a**,**b**). DP-1 cell-derived MVs are labeled with PKH67 and cellular uptake by dTHP-1 cells were observed by fluorescent microscopy as described in “Methods”. The cells were pretreated with or without nystatin or MBCD to suppress lipid raft mediated endocytosis or to achieve cholesterol depletion from culture medium. Data represent means ± SD of at least two independent experiments with identical results. Statistical analyses were performed by one-way ANOVA, followed by Dunnett’s test (**b**). *p < 0.05, **p < 0.01, ***p < 0.001. significant difference versus dTHP-1 cells stimulated with MVs in the absence of nystatin or MBCD (**b**).

As mentioned above, it is very unlikely that a signaling molecule alone is secreted from a certain cell type in its intact form and transferred to another target cell. We, therefore, hypothesized that important signaling molecules may be packed and transported inside the extracellular microparticles. To test this possibility, we first separated the contents of cell culture supernatants from dental pulp cells using different pore-size filters and found that DPIT activity was completely lost in a fraction with a particle size below 220 nm (Fig. 7a). The most abundant microvesicles from dental pulp cells isolated by ultra-centrifugation had a particle size of 230–250 nm, and round-shaped morphology of microvesicles was identified by transmission electron microscopy (TEM) analysis (Fig. 7b,c). To confirm the activity of these vesicles, we compared the TNF-α-inducing activity of these particles with those of microvesicles derived from Hek293 cells (Hek293-MVs) and MCF7 cells (MCF7-MVs) isolated in a similar manner, and confirmed that only dental pulp cell-derived microvesicles (DP-1-MVs and priDPC-1-MVs) exhibited TNF-α inducing activity at both the gene and protein levels (Fig. 7d,e). Next, we examined MAP kinase and NF-κB activation in dTHP-1 cells stimulated with isolated microvesicles, and confirmed increased phosphorylation of these signaling molecules from 30 min after the stimulation with DP-1-MVs and priDPC-1-MVs but not Hek293-MVs or MCF7-MVs (Fig. 8a). Furthermore, pre-treatment of dTHP-1 cells with NF-κB, ERK1/2, p38, and JNK1/2 inhibitors led to reduced TNF-α gene and protein expression after DP-1-MVs stimulation. These reductions were marked, especially when the cells were pre-treated with the JNK inhibitor, while an inhibitor for receptor-interacting protein kinase 1 **(**RIPK1) had no inhibitory effect, suggesting that JNK is an important signaling molecule that mediates TNF-α induction (Fig. 8b,c). Further, we stimulated DP-1 cells with several concentrations of DP-1-MVs in fresh serum-free medium to clarify the autocrine effects of dental pulp cell-derived microvesicles and found that the gene expression levels of IL-6, MCP-1, and TNF-α were unchanged in DP-1 cells in contrast to the dTHP-1 cells, in which these gene expression levels were drastically increased by the stimulation with the DP-1-MVs (Supplementary Fig. 5). These results indicated that DP-1-MVs did not have autocrine effect. We then labeled dental pulp DP-1-MVs with a fluorescent dye and observed their cellular uptake by dTHP-1 cells. After 100 min of incubation with microvesicles, cellular uptake was observed to be initiated (Fig. 9a). Cellular intake appeared to be inhibited by nystatin treatment, but not by methyl-β-cyclodextrin (MBCD) treatment. In fact, TNF-α production from dTHP-1 cells stimulated with DP-1-MVs for 2 hrs was completely inhibited by nystatin treatment, an inhibitor of membraneous cholesterol-mediated endocytosis, but not by MBCD treatment, a reagent for cholesterol depletion, suggesting that microvesicles were incorporated by dTHP-1 cells and promoted TNF-α synthesis (Fig. 9b). Further, to exclude the possibility that cholesterol depletion might disrupt the integrity of microvesicles, we incubated DP-1-MVs with the equivalent concentration of nystatin and MBCD utilized for treatment of dTHP-1 cells and found that the leakage of microvesicle-proteins such as HSP90α and GAPDH into the solution after 100 min incubation was not obvious when treated with either nystatin or MBCD unlike the DP-1-MVs treated with TritonX-100 (1%), which induced lysis of microvesicles (Supplementary Fig. 6). Certain Toll like receptors (TLRs) have been suggested to act as receptors for exosomal or microvesicle-derived miRNA^11,12^. We therefore examined the involvement of these receptors in cellular uptake of the microvesicles. However, neither gene silencing nor selective inhibitors of TLR signaling and their down-stream molecules such as MyD88, TRAF6, TAK1, RIPK1, and TRADD resulted in decreased TNF-α secretion from dTHP-1 cells after stimulation with DP-1-MVs (Supplementary Fig. 7).

**Figure 10:**
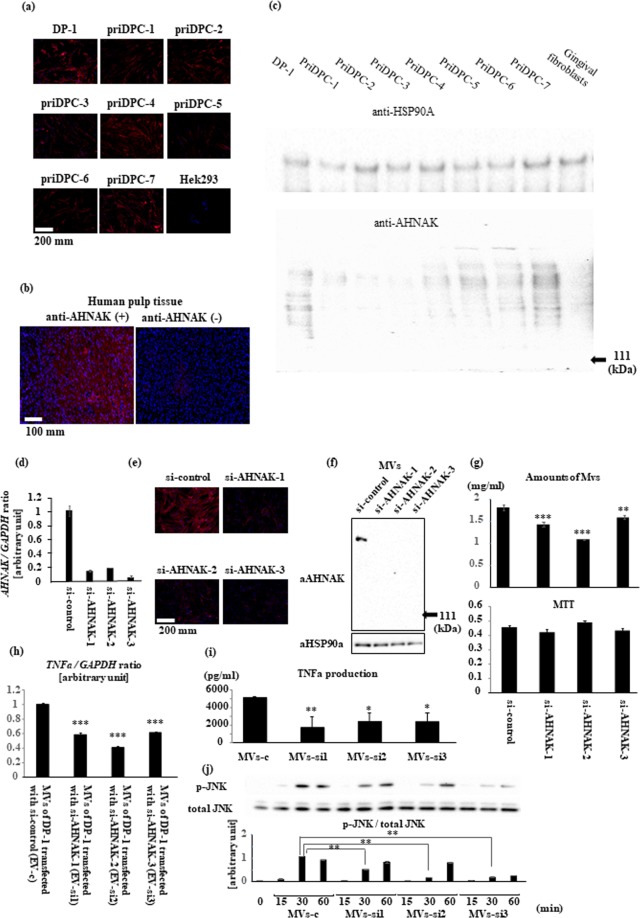
Giant molecule, AHNAK, promotes DPIT activity. (**a**–**c**) Giant molecule AHNAK was detected in DP-1 and PriDPCs (**a**), dental pulp tissue (**b**), and from PriDPC supernatants (**c**). (**d**–**g**) AHNAK gene silencing by 3 independent siRNAs (**d**: mRNA expression; **e**: immunohistochemistry of AHNAK in DP-1 cells; **f**: AHNAK detection in MVs from transfected DP-1 cells) inhibits MVs secretion (**g**: upper) and the effect was not brought about by an alteration in cell numbers (**g**: lower) as revealed by MTT assays. (**h**–**j**) dTHP-1 cells showed significantly reduced TNF-α gene expression (**h**), TNF-α production (**i**), and JNK activation (**j**) when the cells were stimulated with MVs from siAHNAK-transfected DP-1 cells compared with the cells stimulated with equal amounts of MVs from si-control transfected DP-1 cells. Data represent means ± SD of at least two independent experiments with identical results. Statistical analyses were performed by one-way ANOVA, followed by Dunnett’s test (**g**–**j**). *p < 0.05, **p < 0.01, ***p < 0.001. significant difference versus DP-1 cells transfected with control siRNA (**g**) or dTHP-1 cells stimulated with the MVs obtained from DP-1 cells transfected with control siRNA (**h**–**j**).

**Figure 11:**
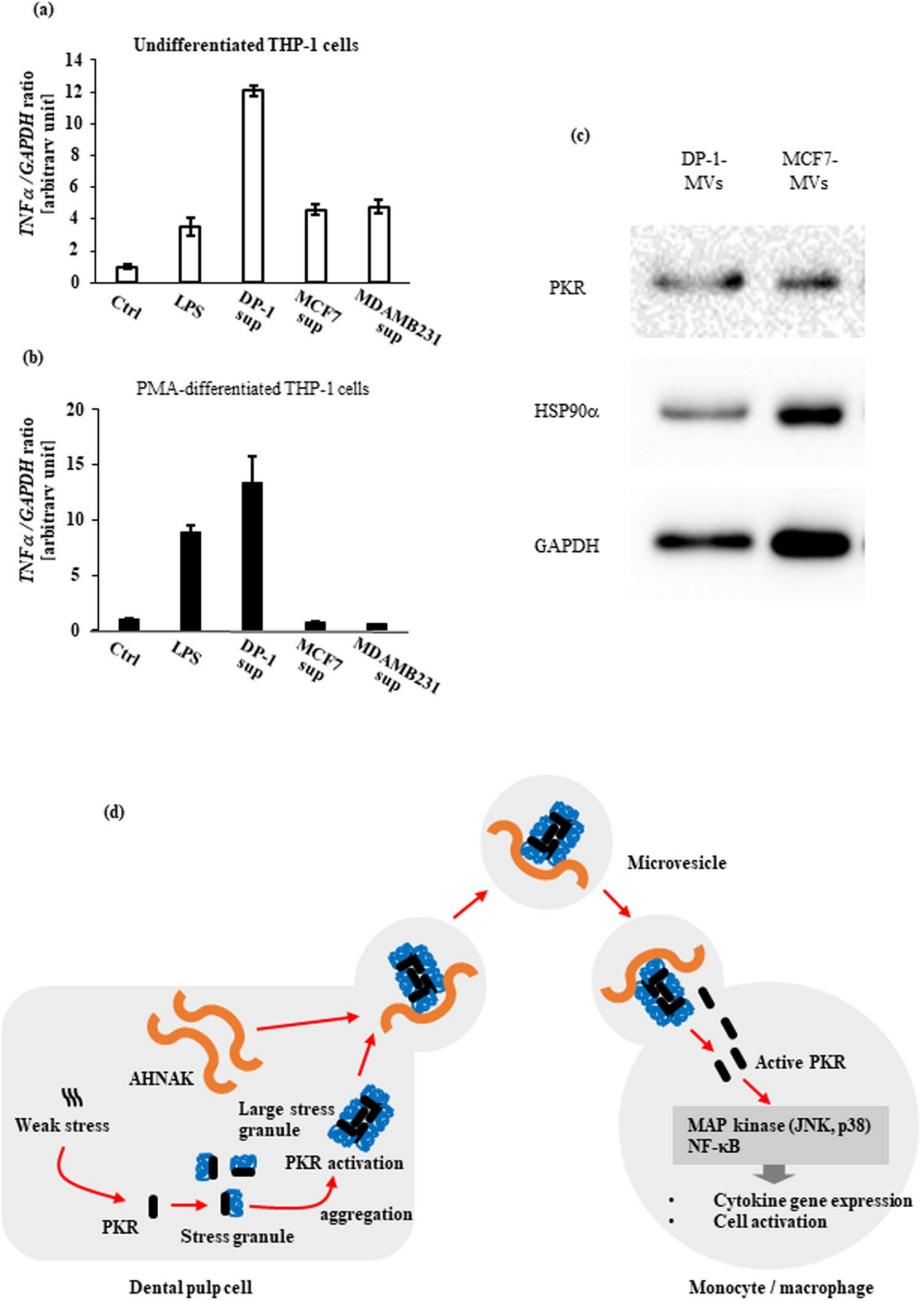
The role of PKR in DPIT activity. (**a**–**c**) Cancer cell-derived supernatants failed to induce TNF-α gene expression in either undifferentiated (**a**) or differentiated (**b**) THP-1 cells, although PKR protein was identified in both DP-1-MVs and MCF7-MVs (**c**). (**d**) Schematic representation of the role of DPTIF in activation of innate immune responses. In dental pulp cells, sustained, weak stress signals constitutively activate interferon-inducible signals, resulting in activation of PKR. Activated PKR and stress granule proteins form stress granule complexes that eventually meet giant protein AHNAK. Large stress granule/AHNAK complexes are released from dental pulp cells in microvesicles. The released microvesicles maintain PKR in an active state, and eventually deliver active PKR into recipient monocytes/macrophages.

Generally, the observed particle size was larger than that of regular exosomes and corresponded to slightly larger microvesicle^13,14^. Therefore, we performed proteomics analyses using the LC-MS/MS method to determine the kinds of molecules included in culture supernatants from DP-1 with DPIT activity, and identified several marker proteins typical of microvesicles such as GAPDH, β-actin, HSPA8, HSPB1, HSPE1, and HSPD1^14^ (Table 2). Furthermore, the most abundantly detected protein in the culture supernatants was neuroblast differentiation-associated protein AHNAK, with a molecular size of 700 kDa. AHNAK is a giant protein associated with pseudopod formation and is often seen in extracellular particles^15–17^. In fact, AHNAK protein was detected in the cytosol of dental pulp cells, but not Hek293 cells (Fig. 10a), and also in dental pulp tissue (Fig. 10b). In addition, AHNAK was detected in culture supernatants from dental pulp cells (Fig. 10c). Thus, we examined the possible roles of this molecule in active molecule transportation. Gene silencing of AHNAK in DP-1 with siRNAs was successful, with significant reduction in microvesicle production (Fig. 10d–g). Treatment of DP-1 with the siRNA resulted in abolition of AHNAK in microvesicles, and led to decreased TNF-α gene expression and protein production from recipient dTHP-1 cells challenged with the equal amounts of these microvesicles (Fig. 10h,j). Similarly, significantly decreased signaling as evaluated by JNK phosphorylation was observed (Fig. 10j). These data suggest that AHNAK had a role in the transportation of DPIT. AHNAK has been detected in exosomes from certain cancer cells such as breast cancer cells^17^. However, we did not detect any TNF-α-inducing activity in supernatants from these cancer cells (Fig. 11a,b). We also investigated the presence of PKR in extracellular vesicles from both dental pulp cells and the breast cancer cells MCF-7, and confirmed its presence in cancer cell-derived microvesicles (Fig. 11c).

**Table 2:** Proteomics analyses of culture supernatants of DP-1 cells and microvesicle marker proteins.

Rank	Score	Names
1	148.02	AHNAK Neuroblast differentiation-associated protein AHNAK
2	51.85	VIM Vimentin
3	37.35	BASP1 Isoform 1 of Brain acid soluble protein 1
11	22.68	ACTB Actin, cytoplasmic 1
33	10.94	GAPDH Glyceraldehyde-2-phosphate dehydrogenase
94	6.08	HSPD1 60 kDa heat shock protein, mitochondrial
139	4.41	HSPA8 Isofoam 1 of Heat shock cognate 71 kDa protein
143	4.33	HSPE1 10 kDa heat shock protein, mitochondrial
232	3.01	HSPB1 Heat shock protein beta-1
251	2.77	HSP90AB1 Heat shock protein HSP 90-beta
290	2.36	CCT2 T-complex protein 1 subunit beta
342	2.04	HSP90AA1 Isoform 2 of Heat shock protein HSP 90-alpha

Because the breast cancer cells used in our experiments also contained PKR in extracellular compartments, we examined the differences between cells with and without DPIT activity. The possibility was that dental pulp cell-derived microvesicles contained activated or at least pre-activated PKR in their compartments without activation of target substrates inside the vesicles and that the active kinase was trans-introduced to the recipient monocyte/macrophage cell line. We carefully re-examined the proteomics data and confirmed the detection of many recently reported stress granule-associated proteins in the culture supernatants from pulp cells (Supplementary Table 1)^18–28^. In particular, eukaryotic translation initiation factor 4 (EIF4), caprin 1, and Ras GTPase-activating protein-binding protein (G3BP), all of which were reported to directly bind to PKR in stress granules^29^, were detected with other stress granule proteins (Table 3). Large stress granules were reported to retain PKR in an active state, but in a non-auto-phosphorylated form^29^. However, we also detected a PKR substrate protein, eukaryotic translation initiation factor 2 (EIF2), in DP-1 supernatant (Table 3). Thus, we treated the culture supernatants with a PKR inhibitor and examined their DPIT activity. Surprisingly, treatment of the culture supernatants with a low concentration of the PKR inhibitor up-regulated DPIT activity (Supplementary Fig. 8). These results suggested that the kinase inhibitor suppressed PKR ejection from stress granule aggregates and subsequent auto-phosphorylation within the vesicles, thereby maintaining PKR in a pre-active state and inhibiting activation of EIF2. Furthermore, as soon as the inhibitor-treated vesicles encountered recipient macrophages, they released considerable amounts of active PKR into the recipient cells. Endogenous PKR activator, a protein activator of interferon-induced PKR, also known as PACT, was reported to be a stress granule protein^30^. Therefore, we examined whether DP-1 supernatants contained PACT in their vesicles. However, we were unable to detect PACT protein in DP-1-MVs, although it was detected in DP-1 lysate (Supplementary Fig. 9). Moreover, down-regulation of endogenous PACT with a siRNA in DP-1 did not result in decreased DPIT activity (Supplementary Fig. 10), suggesting that a “factor X” other than PACT was acting as an endogenous activator of PKR inside the large stress granules^29^. Future study is needed to identify “factor X” and, in fact, MCF-7 cells as well as MCF-7-MVs appear to be good tools to search for the candidate molecule of “factor X” by utilizing exogenous overexpression and inhibition of endogenous expression strategies since MCF7 cell-derived vesicles appear to contain other necessary components than “factor X”. Based on these experiments, the following mechanism is proposed. In dental pulp cells, a sustained weak stress signal is evoked, resulting in activation of a series of interferon-inducible genes and PKR. Activated PKR continuously promotes the formation and aggregation of stress granules containing active PKR, and the dental pulp cells release stress granule aggregates together with AHNAK into microvesicles. These microvesicles maintain PKR in a pre-active state inside the stress granule aggregates and eventually inject PKR into target cells such as monocytes and macrophages. PKR becomes auto-phosphorylated just after ejection from the stress granule aggregates and exhibits kinase activity. A scheme explaining this cascade is shown in Fig. 11d.

**Table 3:** Stress granule-associated proteins which possibly bind to PKR.

Proteins	Description	Score
G3BP1	Ras GTPase-activating protein-binding protein 1	1.4
G3BP2	Ras GTPase-activating protein-binding protein 2	1.52
EIF4G1	eukaryotic translation initiation factor 4 gamma 1 isoform 1	3.65
EIF4EBP1	Eukaryotic translation initiation factor 4E-binding protein 1	4.55
EIF4EBP2	Eukaryotic translation initiation factor 4E-binding protein 2	0.15
CAPRIN1	Caprin-1	0.72
EIF2S2	Eukaryotic translation initiation factor 2 subunit 2	3.22
EIF2A	Eukaryotic translation initiation factor 2A	0.09
EIF2S1	Eukaryotic translation initiation factor 2 subunit 1	0.09

In conclusion, an extremely powerful endogenous TNF-α inducer from macrophages was discovered. The TNF-α-inducing activity of this factor was superior to that of other known TNF-α inducers like bacterial endotoxin. Furthermore, we showed that this strong innate inflammatory activator was transported via extracellular particles. If similar phenomena are observed in serious autoimmune- or neuro-degenerative diseases, the current findings will greatly contribute to our understanding for the patho-physiology of these diseases.

## Methods

### Ethics statement

Human dental pulp cells and tissues were collected in compliance with the Hiroshima University ethical guidelines for epidemiological research. All experimental procedures were approved by the Committee of Research Ethics of Hiroshima University (Permit Number: D88-3). Informed consent was obtained from each donor prior to the tissue collection.

### Reagents

LPS B4 (L2630), NFκB inhibitors (BAY 11-70, parthenolide), PKC inhibitor (GF109203X), PKR inhibitors (C-16), JNK inhibitor (SP600125), CamKII inhibitor (KN-93), nystatin, and MBCD were purchased from Sigma (St. Louis, MO). Pam3CSK4 and PKR inhibitor (2-Aminopurine: 2-AP) were purchased from Invivogen (San Diego, CA). ERK inhibitor (FR180204) and p38 inhibitor (SB203580) were purchased from TOCRIS Bioscience (Bristol, UK). RIPK1 inhibitor (necrostatin-1) was purchased from Cayman Chemical (Ann Arbor, MI). Recombinant IL-6 and MCP-1 were purchased from R&D Systems (Minneapolis, MN).

### Cells

The preparation of immortalized dental pulp cells (DP-1) cells was described previously^2^. Human breast cancer MCF-7 cells and human monocytic leukemia THP-1 cells were purchased from RIKEN Cell Bank (Tsukuba, Japan). Human breast cancer MDAMB-231 cells were purchased from American Type Culture Collection (Manassas, VA). Primary dental pulp (PriDPC) cells were isolated from outgrowth of dental pulp tissue explanted from teeth extracted for orthodontic reason or pericoronitis^31^. DP-1, PriDPC, MCF-7, and MDAMB231 cells were maintained in DMEM (Life Technologies, Carlsbad, CA) supplemented with 100 U/ml penicillin, 100 μg/ml streptomycin, and 10% fetal bovine serum (FBS, Biowest, France). THP-1 cells were maintained in RPMI 1640 medium (Nacalai Tesque, Kyoto, Japan) containing 10% heat-inactivated FBS (HI-FBS), 2 mM GlutaMAX^TM^ (Gibco, Thermo Fisher Scientific, Waltham, MA) and antibiotics. THP-1 cells were seeded in 24-well plates at a density of 4 × 10^5^ cells/well and stimulated with 100 nM PMA (Sigma Aldrich, St. Louis, MO) for 24 h. The cells were then washed twice with PBS and resuspended in PMA-free RPMI 1640 medium containing 10% HI-FBS. After a further 24 h of incubation, the cells attached to the dishes were used as PMA-differentiated macrophages. Human CD14+ peripheral blood-derived monocytes (PBMCs) were purchased from Precision Bioservices (Frederick, MD). PBMC-derived macrophages were differentiated as described previously^32^

### Quantitative PCR (qPCR) analysis

RNA was isolated from DP-1 cells, priDPC cells, THP-1 cells, and PBMCs by RNAiso plus (Takara Bio Inc., Otsu, Japan). Using ReverTra Ace qPCR RT master mix with gDNA remover (Toyobo Life Science, Osaka, Japan), 0.5 μg of total RNA was reverse-transcribed into cDNA utilizing mixed primers. qPCR was performed with a KAPA SYBR Fast qPCR Kit (Kapa Biosystems, Woburn, MA). GAPDH was evaluated as an internal reference control. The PCR primer pairs are shown in Supplementary Table 2.

### TNF-α, IL-1β, IL-6, and MCP-1 measurement

Differentiated THP-1 cells were washed with serum-free medium and stimulated with supernatants from DP-1cells or priDPC cells, matrix vesicles from DP-1, priDPC, HEK293, and MCF-7 cells, Pam3CSK4, and LPS. After incubation for specified periods, the supernatants were collected and measured for their amounts of TNF-α, IL-1β, IL-6, and MCP-1 using a Human TNF-α Quantikine ELISA Kit (DTA00; R&D Systems), Human IL-1β/IL-1F2 Quantikine ELISA Kit (DLB50; R&D Systems), Human IL-6 Quantikine ELISA Kit (D6050; R&D Systems), and Human CCL2/MCP-1 Quantikine ELISA Kit (DCP00; R&D Systems), respectively, in accordance with the manufacturer’s instructions.

### Cell attachment and morphology studies

Frequencies of adherent cells and morphological changes were observed using an inverted phase-contrast light microscope (DMIRB; Leica, Bensheim, Germany). Floating undifferentiated THP-1 cells were seeded in 6-well plates at a density of 1 × 10^6^ cells/well, and stimulated with or without 100 nM PMA in RPMI growth medium or supernatants from DP-1 cells. The plates were incubated in a humidified atmosphere (37°C, 5% CO_2_) for specified periods, and the cells were washed twice with PBS before micrographs were taken.

### Proteomics analyses

Conditioned medium (total: 10 L) was collected from immortalized dental pulp cells (DP-1). The conditioned medium was concentrated using a C18 preparative cartridge and fractions were eluted with acetonitrile. Following evaporation, the fractions were subjected to LC-MS/MS analyses (Filgen Ltd., Nagoya, Japan).

### Microarray analyses

mRNAs were isolated from sub-confluent immortalized dental pulp cells, primary dental pulp cells and control gingival fibroblasts. Equal amounts (10 μg) of the mRNAs were subjected to microarray analyses using Agilent DNA chips. Data analyses were performed as described previously^33^.

### Matrix vesicle purification

Microvesicle-deprived serum was generated by filtration through an 220 nm filter (Millipore, Billerica, MA). DP-1, priDPC-1, HEK293, and MCF-7 cells were maintained until they reached 80% confluency and cultured in fresh medium containing microvesicle-deprived serum for 24 h. On the following day, the supernatants were collected, centrifuged at 2000 rpm for 20 min to remove debris and large apoptotic bodies, and filtered through an 800-nm filter (Millipore). Matrix vesicles were sedimented by centrifugation at 16,000 × g for 45 min. washed twice with DPBS, and resuspended in DPBS containing 0.3 mM EDTA. The amounts of matrix vesicles were quantified by a QuantiPro BCA Assay Kit (Sigma-Aldrich). Dynamic light scattering measurements were performed by UBE Scientific Analysis Laboratory (Tokyo, Japan) with a Zetasizer Nano ZS (Malvern Instruments Ltd., Malvern, UK). Matrix vesicles were suspended in PBS. The machine settings were reflective index of 1.334 and viscosity (cP) of 0.8900 at room temperature. The intensities were recorded three times. Transmission electron microscopy (TEM) analyses were performed by Hanaichi Ultrastructure Research Institute (Okazaki, Japan). The microvesicles in PBS were placed on carbon-film grids for 2 min, from which excess PBS was removed by touching one end of the grid with the filter paper. Then, the grid was touched onto a drop of 2% uranyl acetate and excess liquid was drained off. The grid was allowed to dry for several minutes and then examined using a JEM-1200 EX microscope (JEOL, Akishima, Japan) at 80 kiloelectron volts.

### Gene silencing using siRNAs

Stealth^TM^ RNAi duplexes, comprising mixtures of three different siRNAs against human AHNAK (HSS149070, HSS149071 and HSS149072) and PACT (HSS112561, HSS189437, and HSS189438) were obtained from Invitrogen Life Technologies. Stealth^TM^ RNAi negative control duplex (Medium GC Duplex; Invitrogen Life Technologies) was used as a control. The RNA sequences for targeting PKR, TLR1, TLR3, TLR4, MyD88, TRAF6, TAK1, RIP1, TNFR, TRADD, and IL1BR by siRNAs were selected by Enhanced siDirect, a web-based target-specific siRNA design software. All siRNAs were generated by RNAi Inc. (Tokyo, Japan). The control siRNA used in the study was provided by RNAi Inc. All RNA sequences used for siRNA duplexes are shown in Supplementary Table 3. The siRNAs were reverse-transfected into DP-1 cells and forward-transfected into differentiated THP-1 cells at final concentrations of 10 and 50 nM, respectively, using Lipofectamine RNAiMAX reagent (Life Technologies) and incubated for 48 h.

### SDS-PAGE

Protein samples were reduced with DTT and loaded onto NuPAGE Bis-Tris gels to detect total and phosphorylated JNK, p-38, and p-65, HSP90A, PKR, and PACT and onto NuPAGE Tris-Acetate gels to detect AHNAK. For immunodetection, gels were transferred onto PVDF membranes. The membranes were blocked with PVDF blocking agent (Toyobo, Osaka, Japan), and then incubated with anti-JNK antibody (#9252; Cell Signaling Technology, Danvers, MA), anti-phospho-JNK antibody (#4668; Cell Signaling Technology), anti-total p-38 antibody (#8690; Cell Signaling Technology), anti-phospho-p-38 antibody (#4511; Cell Signaling Technology), anti-total p65 antibody (#8242; Cell Signaling Technology), anti-phospho-p65 antibody (#3033, Cell Signaling Technology), anti-HSP90α antibody (GTX109753; GeneTex, Irvine, CA), anti-AHNAK antibody (ab178317; Abcam, Cambridge, MA), anti-PKR antibody (ab32506; Abcam), or anti-PACT antibody (ab75749; Abcam). The immunoreactive proteins were identified using a Westernbright Quantum Kit (K-12042-D20; Advansta, Menlo Park, CA).

### Uptake analysis

Matrix vesicles were stained with PKH67 fluorescent dye (Sigma-Aldrich). washed with DPBS, and centrifuged at 16,000 × g for 45 min. Differentiated THP-1 cells were pretreated with nystatin or MBCD for 30 min. Next, 80 ng of PKH67-labeled matrix vesicles was incubated with 2 × 10^5^ primed THP-1 cells for 100 min. Uptake of PKH67-labeled matrix vesicles was analyzed with an FV1000-D (Olympus, Tokyo, Japan).

### Immunofluorescence analyses

DP-1 and priDPC cells were seeded into 8-well non-coated glass chamber slides (SCS-N08; Matsunami, Osaka, Japan), fixed with 3.7% formaldehyde for 10 min, permeabilized with 0.5% Triton X-100 in DPBS for 5 min, blocked with 1% BSA in DPBS for 1 h at room temperature, and incubated with anti-AHNAK antibody (1:200 dilution) overnight at 4°C. On the following day, the cells were incubated with Cy3-conjugated goat anti-rabbit secondary antibody (1:1000 dilution) for 1 h at room temperature and embedded with SlowFade™ Diamond Antifade Mountant containing DAPI (Life Technologies). Immunofluorescence signals were observed using an FV1000-D microscope (Olympus). Human dental pulp tissues dissected from healthy teeth extracted for orthodontic purposes were fixed with 3.7% formaldehyde for 24 h, sequentially immersed in 15% sucrose in PBS overnight and 30% sucrose overnight, embedded in OCT compound, and sectioned at 10-μm thickness. Rehydrated sections were treated as described above to detect AHNAK expression in human dental pulp tissues.

### MTT assay

MTT cell proliferation assays were performed to calculate the numbers of surviving cells using an MTT Kit (Roche, Basel, Switzerland) following the manufacturer’s protocol. The solubilized formazan product was spectrophotometrically quantified by measuring the absorbances at 575 nm and 650 nm on a microtiter plate reader (Multiskan).

## Supplementary information

Data set 1
