# Supplementary material for: Dental pulp cell-derived powerful inducer of TNF-α comprises PKR containing stress granule rich microvesicles
Source: Sci Rep. 2019 Mar 7;9:3825. doi: 10.1038/s41598-019-40046-2 (PMC6405945; doi:10.1038/s41598-019-40046-2)
Supplement: Supplementary file 1 — Data set 1 [file 41598_2019_40046_MOESM1_ESM.docx]

**Supplementary files**

**Dental pulp cell-derived powerful inducer of TNF-α comprises PKR containing stress granule rich microvesicles**

Shigeki Suzuki ^a, c^, Takao Fukuda ^b^, Shintaro Nagayasu ^c^, Jun Nakanishi ^c^, Kazuma Yoshida ^c^, Shizu Hirata-Tsuchiya ^c^, Yuki Nakao ^b^, Tomomi Sano ^b^, Akiko Yamashita ^b^, Satoru Yamada ^a^, Kouji Ohta ^d^ , Hideki Shiba ^c^, Fusanori Nishimura ^b, *^

a. Department of Periodontology and Endodontology, Tohoku University Graduate School of Dentistry, 4-1 Seiryo-machi, Aoba-ku, Sendai, 980-8575, Japan

b. Section of Periodontology, Department of Oral Rehabilitation, Kyushu University Faculty of Dental Science, 3-1-1 Maidashi, Higashi-ku, 812-8592, Japan

c. Department of Biological Endodontics, Graduate School of Biomedical and Health Sciences, Hiroshima University, 1-2-3 Kasumi, Hiroshima, 734-8553, Japan

d. Department of Oral & Maxillofacial Surgery, Graduate School of Biomedical and Health Sciences, Hiroshima University, 1-2-3 Kasumi, Hiroshima, 734-8553, Japan

**Supplementary Figure 1. Exogenous addition of recombinant IL-6 and MCP-1 is unable to induce TNF-α production in dTHP-1 cells.**

dTHP-1 cells are stimulated with recombinant IL-6 and MCP-1 (1, 10, and 100 ng/ml) for 24 hrs and the amounts of TNF-α in the supernatants are evaluated with specific ELISA kits. dTHP-1 cells stimulated with the indicated concentrations of IL-6 and MCP-1 are not induced toward TNF-α production unlike the case for cells treated with LPS.

**Supplementary Figure 2. DP-1 sup induced cell growth of undifferentiated THP-1 cells.**

Floating undifferentiated THP-1 (5 × 10^4^ cells) in 100 μl of culture medium are seeded into a 96-well culture plate, and stimulated with 100 nM PMA or incubated with DP-1 supernatant (DP-1). Next, 10 μl of WST-8 solution are added to each well (including the control wells), at 6, 12, 24, and 48 hrs. Cells are incubated for an additional 1 hr at 37°C, then absorbance at 450 nm is measured, with a reference reading at 590 nm. Values are presented as mean ± S.D., n = 5. Statistical analyses are performed using a two-tailed unpaired Student's t-test: *p < 0.05; **p < 0.01.

**Supplementary Figure 3. PKR expression in DP-1, PriDPC-1, gingival fibroblast cell, which used for microarray analyses.**

As similar to microarray analyses, PKR expression level in DP-1 and PriDPC-1 is significantly higher than that in gingival fibroblast cells. Data represent means ± SD of at least two independent experiments with identical results. **p < 0.01, ***p < 0.001 significant higher than gingival fibroblast cells.

**Supplementary Figure 4. LPS activates JNK, p-38, and p65 signaling in dTHP-1 cells.**

dTHP-1 cells are stimulated with various concentrations of LPS for 1 hr and the activation of JNK, p-38, and p65 signaling are monitored utilizing their phosphor-specific antibodies. Saturated area is colored red.

**Supplementary Figure 5. DP-1-MVs are unable to induce IL-6, MCP-1, and TNF-α expression in DP-1 cells.**

Serum-starved DP-1 cells and dTHP-1 cells are washed twice with serum-free medium and then stimulated with several concentrations of DP-1-MVs for 2 hrs and then total RNAs are collected to analyze the gene expression levels of IL-6 (A), MCP-1 (B), and TNF-α (C).

**Supplementary Figure 6. The integrity of MVs after cholesterol deprivation by nystatin and MBCD.**

MVs are treated with nystatin, MBCD, or 1% TritonX-100 at indicated concentrations for 100 min and then samples are centrifuged to separate MVs from the solution. The amounts of HSP90α and GAPDH, major components of MVs, are comparatively evaluated. MVs treatment with 1% TriotonX-100 is performed for complete lysis. Triton = TritonX-100.

**Supplementary Figure 7. TLRs and their down-stream signaling molecules are dispensable for dental pulp cell microvesicle-dependent TNF**-α **expression and TNF-α secretion in dTHP-1 cells.**

（A）dTHP-1 cells are transfected with siRNAs targeting various TLRs and their downstream signaling molecules, and effective knock down of the target genes is confirmed. (B) The amounts of TNF-α production in dTHP-1 cells transfected with the siRNA do not differ significantly from those in cells transfected with the control siRNA after stimulation with DP-1-MVs.

**Supplementary Figure 8. Treatment of DP-1 sup with a low concentration of PKR inhibitor C-16 leads to enhanced TNF-α induction activity in dTHP-1 cells.**

**Supplementary Figure 9. PACT identification in cell lysates, but not MVs, from DP-1, MCF-7, and MDAMB-231 cells.**

**Supplementary Figure 10. Knockdown of PACT is unable to reduce the TNF**-α**-inducing ability of DP-1 sup.** n.s.: not significant.

| **Supplementary Table 1. Stress granule-associated proteins detected by proteomics analysis** | | | |
| --- | --- | --- | --- |
|  |  |  |  |
| **Protein detected** | **Description** | **score** | **Reference** |
| ABCF1 | ATP Binding Cassette Subfamily F Member 1 | 0.44 | 18 |
| ACTBL2 | Beta-actin-like protein 2 | 22.68 | 19 |
| ADAR1 | Adenosine Deaminase, RNA Specific | 0.26 | 19 |
| ANKRD17/MASK2/GTAR | Ankyrin Repeat Domain 17 | 0.36 | 18 |
| ANXA1 | Annexin A1 | 5.21 | 19 |
| ASCC1 | Armadillo Repeat Containing 6 | 0.89 | 18 |
| ATP6V1G1/ATP6G | ATP synthase subunit alpha, mitochondrial | 6.73 | 19 |
| BAG3 | Ataxin 2 Like | 3.72 | 18, 19 |
| BANF1 | BAG family molecular chaperone regulator 3 | 5.02 | 19 |
| BCCIP | Barrier-to-autointegration factor | 0.25 | 19 |
| BICC1 | BCL2 Associated Transcription Factor 1 | 0.43 | 18 |
| Caprin-1 | Adenylyl cyclase-associated protein 1 | 0.72 | 19 |
| MLN51/BTZ | Calcium-regulated heat stable protein 1 | 1.8 | 19 |
| CCDC85C | Coiled-Coil Domain Containing 124 | 9.24 | 18 |
| CDC73 | Cell division cycle 5-like protein | 0.68 | 19 |
| CUGBP1 | CDV3 Homolog | 9.79 | 18 |
| CHCHD3 | Cofilin-1 | 8.89 | 19 |
| CNOT1/CCR4 | Calponin-3 | 0.37 | 19 |
| Carboxypeptidase B2 | Coronin-1B | 2.01 | 19 |
| CSTF1 | Cystatin B | 2.7 | 18 |
| CWC22 | CTTNBP2 N-terminal-like protein | 0.08 | 19 |
| PRTB | DAZ-associated protein 1 | 0.12 | 18, 19 |
| DCP1b | Decapping mRNA 1a | 0.46 | 18, 19 |
| DDX19A | DEAD-Box Helicase 1 | 2.8 | 18, 19 |
| DDX21 | ATP-dependent RNA helicase DDX19A | 0.42 | 19 |
| DEAD box protein 3 | Nucleolar RNA helicase 2 | 2.79 | 19 |
| DPYSL3 | Dihydropyrimidinase-related protein 2 | 1.23 | 19 |
| DUSP12/YVH1 | E3 ubiquitin-protein ligase DTX3L | 4 | 19 |
| Cytoplasmic Dynein Light Polypeptide | Dynein Cytoplasmic 1 Heavy Chain 1 | 3.7 | 20 |
| EIF1 | Eukaryotic translation initiation factor 1 | 6.43 | 18, 19 |
| EIF2A | Eukaryotic Translation Initiation Factor 2A | 4.47 | 18 |
| EIF2A subunit 2 | Eukaryotic Translation Initiation Factor 2 Subunit Alpha | 0.09 | 19 |
| EIF3A | Eukaryotic Translation Initiation Factor 2 Subunit Beta | 4.36 | 19 |
| EIF3B | Eukaryotic Translation Initiation Factor 3 Subunit A | 4.36 | 18, 19 |
| EIF3C | Eukaryotic Translation Initiation Factor 3 Subunit B | 2 | 18, 19 |
| EIF3D | Eukaryotic Translation Initiation Factor 3 Subunit C | 1.34 | 18 |
| EIF3G | Eukaryotic translation initiation factor 3 subunit F | 1.16 | 19 |
| EIF3H | Eukaryotic translation initiation factor 3 subunit G | 1.16 | 18, 19 |
| EIF3J | Eukaryotic translation initiation factor 3 subunit I | 0.36 | 19 |
| EIF3K | Eukaryotic translation initiation factor 3 subunit J | 0.36 | 18, 19 |
| EIF4A1 | Eukaryotic translation initiation factor 3 subunit M | 0.87 | 19 |
| EIF4E | Eukaryotic translation Initiation factor 4B | 9.4 | 18, 19 |
| EIF4G2 | Eukaryotic Translation Initiation Factor 4G1 | 3.65 | 18, 19 |
| EIF4H | Eukaryotic Translation Initiation Factor 4G3 | 0.34 | 18 |
| EIF5A | Eukaryotic translation Initiation factor 4H | 0.34 | 18, 19 |
| HuR | Eukaryotic Translation Initiation Factor 5A | 10.53 | 21 |
| FAM120C | Constitutive coactivator of PPAR-gamma-like protein 1 | 1.55 | 18, 19, 26 |
| FNDC3B | Fragile X Mental Retardation 1 | 2.37 | 18, 19, 26 |
| G3BP2 | G3BP Stress Granule Assembly Factor 1 | 1.4 | 18, 19, 26 |
| GLE1 | GRB10 Interacting GYF Protein 2 | 0.06 | 18 |
| H1F0 | G1 To S Phase Transition 1 | 3.13 | 18 |
| HMGB3 | High mobility group protein HMG-I/HMG-Y | 3.06 | 19 |
| HnRNPA1 | Non-histone chromosomal protein HMG-14 | 3.31 | 19 |
| HnRNPA2/B1 | Heterogeneous Nuclear Ribonucleoprotein A1 | 6.54 | 18, 19 |
| HNRNPA3 | Heterogeneous Nuclear Ribonucleoprotein A2/B1 | 5.69 | 18, 19 |
| HNRNPD | Heterogeneous nuclear ribonucleoprotein A/B | 5.69 | 18, 19, 26 |
| HNRNPDL | Heterogeneous nuclear ribonucleoprotein D | 9.71 | 18 |
| HNRNPF | Heterogeneous nuclear ribonucleoprotein D-like | 0.25 | 18 |
| HNRNPK | Heterogeneous nuclear ribonucleoprotein H3 | 2.71 | 18 |
| HNRNPUL1 | Heterogeneous Nuclear Ribonucleoprotein K | 8.3 | 19 |
| HSBP1 | Heterogeneous nuclear ribonucleoprotein U-like protein 2 | 1.38 | 19 |
| HSP90 | Heat Shock Factor Binding Protein 1 | 0.22 | 18 |
| HSPB8 | Heat Shock Protein Family B (Small) Member 1 | 3.01 | 19 |
| Huntingtin | 60 kDa heat shock protein, mitochondrial | 6.08 | 18, 19 |
| IPO7 | Interleukin Enhancer Binding Factor 3 | 1.09 | 22 |
| KHDRBS3 | KH RNA Binding Domain Containing, Signal Transduction Associated 1 | 0.91 | 19 |
| KIF23 | Kinesin Family Member 13B | 2 | 18 |
| LINE1 ORF1p | Karyopherin Subunit Beta 1 | 0.56 | 19, 27 |
| LARP1B | La-related protein 1 | 2.56 | 19 |
| LIM And SH3 Protein 1/MLN50 | La Ribonucleoprotein Domain Family Member 4B | 0.05 | 18 |
| LBR | LIM And SH3 Protein 1 | 14.41 | 18 |
| LPP | Prelamin-A/C | 14.34 | 19 |
| LSM14B | LSM14A, mRNA Processing Body Assembly Factor | 0.1 | 18, 19, 26 |
| LSM3 | Protein LSM14 homolog B | 0.1 | 18, 19, 26 |
| LUC7L | U6 snRNA-associated Sm-like protein LSm3 | 0.61 | 19 |
| JNK1 | Mitogen-activated protein kinase kinase kinase kinase 4 | 2.13 | 19 |
| MCM5 | DNA replication licensing factor MCM4 | 0.05 | 19 |
| FAM195B/GRAN2 | Methionyl Aminopeptidase 2 | 0.24 | 18 |
| MKRN2 | Antigen KI-67 | 12.31 | 19 |
| NXF1/MEX67/TAP | Nexilin | 0.1 | 19 |
| NonO | Nucleolar And Coiled-Body Phosphoprotein 1 | 1.81 | 18 |
| NOP58 | Non-POU Domain Containing Octamer Binding | 1.38 | 19 |
| NUPL2 | Nuclear fragile X mental retardation-interacting protein 2 | 0.2 | 18, 19, 26 |
| OTUD4/HIN1 | Oxysterol Binding Protein Like 9 | 2.95 | 18 |
| PABPC4 | Poly(A) Binding Protein Cytoplasmic 1 | 6.17 | 18, 19, 26 |
| Palladin | Serine/threonine-protein kinase PAK 4 | 0.31 | 18, 19 |
| PCBP1/HNRNPE1 | PRKC apoptosis WT1 regulator protein | 3.12 | 19 |
| PCBP2/HNRNPE2 | Poly(RC) Binding Protein 1 | 0.08 | 18 |
| PDAP1 | Proliferating cell nuclear antigen | 0.18 | 19 |
| PDCD4 | PDGFA Associated Protein 1 | 3.64 | 18 |
| PDLIM1 | Protein Disulfide Isomerase Family A Member 3 | 0.09 | 18 |
| PDLIM4 | PDZ and LIM domain protein 1 | 5.47 | 19 |
| PDLIM5 | PDZ and LIM domain protein 4 | 4.51 | 19 |
| PDS5B | PDZ and LIM domain protein 5 | 1.87 | 19 |
| Profilin 1 | Prefoldin subunit 4 | 0.09 | 19 |
| PPP1R10 | Protein Phosphatase 1 Regulatory Subunit 8 | 0.45 | 18 |
| PPP2R1B | Serine/threonine-protein phosphatase 2A 65 kDa regulatory subunit A alpha isoform | 0.18 | 19 |
| PKC-ɑ | Protein Kinase AMP-Activated Catalytic Subunit Alpha 2 | 2.6 | 23 |
| PSP1 | 26S proteasome non-ATPase regulatory subunit 2 | 6.58 | 19 |
| PTBP3 | Polypyrimidine tract-binding protein 1 | 4.89 | 18 |
| Pumilio-2 | Pumilio homolog 1 | 0.13 | 18, 19, 26 |
| PMR1 | Peroxidasin Like | 0.08 | 18 |
| RNH1 | Ribonuclease inhibitor | 2.21 | 18 |
| 40S Ribosomal Protein S3 | 40S Ribosomal Protein S3 | 0.5 | 24 |
| PAI-1/Serpin E1 | Serpine Family E Member 1 | 5.51 | 25 |
| SFRS3 | Serine/arginine-rich splicing factor 3 | 4.75 | 19 |
| SMAP2 | Small ArfGAP2 | 0.06 | 18 |
| SNTB2 | Beta-2-syntrophin | 0.43 | 19 |
| SPATS2/SPATA10/SCR59 | Spermatogenesis Associated Serine Rich 2 | 0.08 | 19 |
| SPECC1L | Cytospin-A | 1.11 | 19 |
| SS18L1/CREST | SS18L1, nBAF Chromatin Remodeling Complex Subunit | 0.05 | 18 |
| Staufen 2 | Staufen Double-Stranded RNA Binding Protein 2 | 0.49 | 18, 19 |
| STRAP | Serine-threonine kinase receptor-associated protein | 2.15 | 19 |
| SUN1 | SUN domain-containing protein 1 | 0.13 | 26 |
| TCEA1 | Transcription elongation factor A protein 1 | 3.19 | 18 |
| THRAP3 | Thyroid Hormone Receptor Associated Protein 3 | 2.77 | 27 |
| TMOD3 | Tropomodulin-3 | 2.01 | 26 |
| TNKS1BP1 | 182 kDa tankyrase-1-binding protein | 9.88 | 28 |
| TPM1 | Tropomyosin alpha-1 chain | 0.08 | 26 |
| TPM2 | Tropomyosin beta chain | 5.27 | 19 |
| TRIP6 | Thyroid receptor-interacting protein 6 | 2.88 | 18 |
| TUBB3 | Tubulin beta-3 chain | 3.52 | 19 |
| TXN | Thioredoxin | 2.9 | 19 |
| TXNDC17 | Thioredoxin Domain Containing 17 | 0.17 | 19 |
| UBAP2L | Ubiquitin-associated protein 2-like | 5.31 | 18, 19, 26 |
| VASP | Vasodilator-stimulated phosphoprotein | 0.51 | 18 |
| YLPM1 | YLP Motif Containing 1 | 2.02 | 19 |
| YTHDF3 | YTH domain family protein 3 | 2.01 | 18, 19, 26 |
| 41701 | 14-3-3 protein eta | 0.16 | 19 |
| 41701 | 14-3-3 protein theta | 2.28 | 19 |
|  |  |  |  |

|  |  |  |  |
| --- | --- | --- | --- |
|  | **Supplementary Table 2. Primers used for qPCR** | | |
|  |  |  |  |
|  | TNF-α | forward | TGCTTGTTCCTCAGCCTCTT |
|  |  | reverse | TGGGGAACTCTTCCCTCTG |
|  | MCP-1 | forward | AGCAAGTGTCCCAAAGAAGC |
|  |  | reverse | GAGTTTGGGTTTGCTTGTCC |
|  | IL-6 | forward | TACATCCTCGACGGCATCTC |
|  |  | reverse | TTTCAGCCATCTTTGGAAGG |
|  | PKR | forward | ACGCTTTGGGGCTAATTCTT |
|  |  | reverse | GAGATGATGCCATCCCGTAG |
|  | AHNAK | forward | ATGTGGACATTTCTGCACCA |
|  |  | reverse | TGAAGTGCATCTCAGGCATC |
|  | TLR1 | forward | TATTGGGCACCCCTACAAAA |
|  |  | reverse | AATGGCAAAATGGAAGATGC |
|  | TLR3 | forward | TGTTTTCACGCAATTGGAAG |
|  |  | reverse | CCGAATGCTTGTGTTTGCTA |
|  | TLR4 | forward | AGAACTGCAGGTGCTGGATT |
|  |  | reverse | AACTCTGGATGGGGTTTCCT |
|  | MYD88 | forward | GACTGCTCGAGCTGCTTACC |
|  |  | reverse | CCTGCTGCTGCTTCAAGATA |
|  | TRAF6 | forward | TGGCATTACGAGAAGCAGTG |
|  |  | reverse | TGGACATTTGTGACCTGCAT |
|  | TAK1 | forward | CTTGGATGGCACCTGAAGTT |
|  |  | reverse | GTTTCCGACGCGTTATCACT |
|  | RIP1 | forward | CACAGAACCCAGGGACTCAT |
|  |  | reverse | TTCATCATCTTCGCCTCCTC |
|  | TNFR | forward | ACCAAGTGCCACAAAGGAAC |
|  |  | reverse | GTTTTCTGAAGCGGTGAAGG |
|  | TRADD | forward | TGCCCAGACTTTTCTGTTCC |
|  |  | reverse | GCCATTTGAGACCCACAGAG |
|  | IL1BR | forward | ACGTTGGGGAAGACATTGTT |
|  |  | reverse | CCAGCTGAAGCCTGATGTTT |
|  | PACT | forward | CTGCTTAGTGAAATTGCCAAGGA |
|  |  | reverse | GAGCCATGACAGACTGTGATG |
|  | GAPDH | forward | TCGTGGAAGGACTCATGACC |
|  |  | reverse | GAGGCAGGGATGATGTTCTG |
|  |  |  |  |

|  |  | **Supplementary Table 3. Target sequences for siRNA duplex** | |
| --- | --- | --- | --- |
|  | Oligo Name | Sense Strand (5'→3') | Antisense Strand (5'→3') |
|  | si-EIF2AK 1st | CGGAAAGACUUACGUUAUUAA | AAUAACGUAAGUCUUUCCGUC |
|  | si-EIF2AK 2nd | GGAUCGACCUAACACAUCUGA | AGAUGUGUUAGGUCGAUCCUC |
|  | si-TLR1 | CAUUCCGCAGUACUCCAUUCC | AAUGGAGUACUGCGGAAUGGG |
|  | si-TLR3 | CUCACUAUGCUCGAUCUUUCC | AAAGAUCGAGCAUAGUGAGAU |
|  | si-TLR4 | GUCACUCGAUGUCAUUCCAAA | UGGAAUGACAUCGAGUGACAA |
|  | si-MYD88 | CUGAGCGUUUCGAUGCCUUCA | AAGGCAUCGAAACGCUCAGGC |
|  | si-TRAF6 | CACGUUCCAUGCUUUUUCAGA | UGAAAAAGCAUGGAACGUGUG |
|  | si-TAK1 | CCUUAUAAUGACGAUUCAUGA | AUGAAUCGUCAUUAUAAGGUU |
|  | si-RIP1 | GGAUCCGUUAACGUUAAUACC | UAUUAACGUUAACGGAUCCUG |
|  | si-TNFR | CCCCUCGAUGUACAUAGCUUU | AGCUAUGUACAUCGAGGGGUU |
|  | si-TRADD | CCUCUCUGUCGGAGGUGAAGC | UUCACCUCCGACAGAGAGGGC |
|  | si-IL1BR | CGUGCCUCUCGGGUAGCAUGG | AUGCUACCCGAGAGGCACGUG |
|  | si-control | GUACCGCACGUCAUUCGUAUC | UACGAAUGACGUGCGGUACGU |
